# Injectable hydrogel loaded with exosomes from hypoxic umbilical cord-derived mesenchymal stem cells alleviates intervertebral disc degeneration by reversing nucleus pulposus cell senescence

**DOI:** 10.1093/rb/rbaf039

**Published:** 2025-05-12

**Authors:** Xin Zhao, Yubo Shi, Zhen Sun, Wei Duan, Le Chang, Benchi Xu, Kangwei Lai, Jingchun Zhang, Buqi Tian, Weidong Tao, Zhenzhou Mi, Mian Zhang, Wenjing Yang, Zhuojing Luo, Zhengxu Ye

**Affiliations:** Department of Orthopaedics, Xijing Hospital, Fourth Military Medical University, Xi’an 710032, P. R. China; Department of Orthopedic Surgery, Xiangyang No.1 People’s Hospital, Hubei University of Medicine, Xiangyang 441000, P. R. China; Department of Orthopaedics, Xijing Hospital, Fourth Military Medical University, Xi’an 710032, P. R. China; Department of Orthopaedics, Xijing Hospital, Fourth Military Medical University, Xi’an 710032, P. R. China; Department of Aerospace Medicine, Fourth Military Medical University, Xi’an 710032, P. R. China; Department of Orthopaedics, The 986th Air Force Hospital, Xijing Hospital, The Fourth Military Medical University, Xi’an 710032, P. R. China; Department of Orthopaedics, Xi’an Medical University, Xi’an 710021, P. R. China; Department of Orthopaedics, Xi’an Medical University, Xi’an 710021, P. R. China; Department of Orthopaedics, Xi’an Medical University, Xi’an 710021, P. R. China; Department of Orthopaedics, Xijing Hospital, Fourth Military Medical University, Xi’an 710032, P. R. China; Department of Orthopaedics, Xijing Hospital, Fourth Military Medical University, Xi’an 710032, P. R. China; Department of Orthopaedics, Xijing Hospital, Fourth Military Medical University, Xi’an 710032, P. R. China; Department of Stomatology, The 986th Air Force Hospital, Xijing Hospital, The Fourth Military Medical University, Xi’an 710032, P. R. China; Department of Orthopaedics, Xijing Hospital, Fourth Military Medical University, Xi’an 710032, P. R. China; Department of Orthopaedics, Xijing Hospital, Fourth Military Medical University, Xi’an 710032, P. R. China

**Keywords:** senescence, intervertebral disc degeneration, umbilical cord-derived mesenchymal stem cells, exosome, hydrogel

## Abstract

Intervertebral disc degeneration is a significant contributor to the development of spinal disorders. Previous studies have shown that the senescence of nucleus pulposus cells can worsen the degradation of intervertebral disks. Therefore, targeting the senescence of nucleus pulposus cells may be a promising therapeutic approach for the treatment of intervertebral disc degeneration. This study investigated the use of exosomes from hypoxic umbilical cord-derived mesenchymal stem cells to reverse nucleus pulposus cells senescence and delay intervertebral disc degeneration progression. MicroRNA sequencing of hypoxic umbilical cord-derived mesenchymal stem cells revealed the presence of functional microRNAs, with the p53 signalling pathway identified as a key factor. To enhance the release time of hypoxic umbilical cord-derived mesenchymal stem cells *in vivo*, hyaluronic acid methacryloyl hydrogel was used to load hypoxic umbilical cord-derived mesenchymal stem cells and create a sustained-release system. This system effectively repaired the degradation of the extracellular matrix, reversed nucleus pulposus cells senescence and alleviated intervertebral disc degeneration progression in a rat model. Overall, this study highlights the potential of hypoxic umbilical cord-derived mesenchymal stem cells in reducing nucleus pulposus cell senescence and suggests the possibility of combining it with a sustained-release system as a novel therapeutic strategy for intervertebral disc degeneration.

## Graphical Abstract

**Figure:**
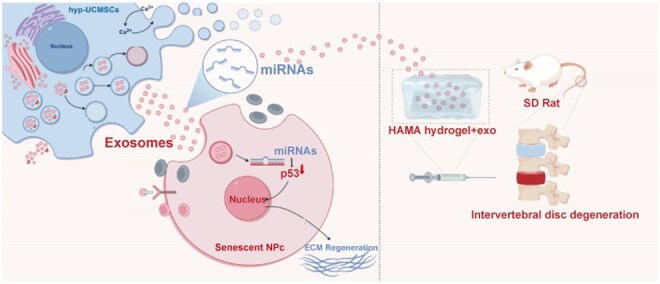

## Introduction

Intervertebral disc (IVD) degeneration (IVDD) is a fundamental disease that affects the quality of life of individuals with a range of spinal disorders [1–3]. Although the exact cause of IVDD remains unclear, researchers believe nucleus pulposus cell (NPC) senescence plays a crucial role in its development [4, 5]. The secretory activities of senescent NPCs exhibit changes referred to as the senescence-associated secretory phenotype (SASP), which disrupts the extracellular matrix (ECM) function of the IVD. These senescent NPCs release catabolic factors and cytokines, leading to the activation of DNA damage and propagation of senescence among the surrounding cells [6, 7]. Consequently, this process exacerbates the degradation of the IVD. Thus, developing therapeutic strategies to reduce NPC senescence and preserve their normal function remains crucial for mitigating IVDD progression.

Owing to the anti-inflammatory effects of mesenchymal stem cells (MSCs), their potential for treating IVDD has been demonstrated [8]. Interestingly, umbilical cord-derived MSCs (UCMSCs) have gained attention for their regenerative ability, anti-inflammatory effects and noninvasive collection [9]. However, the challenging IVD microenvironment, characterized by constant loading, long-lasting oxidative stress and low nutrition, poses a survival challenge for transplanted UCMSCs. Recent evidence suggests that the therapeutic effects of MSCs arise from paracrine effects [10, 11]. Studies have shown that MSC-derived exosomes (MSC-exos) have the ability to enhance cell migration and stimulate the secretion of anti-inflammatory factors [12]. Furthermore, studies have shown that MSC-exos alleviate cell senescence in various tissues [13, 14]. These findings demonstrate that MSC-exos could serve as a novel therapeutic approach for addressing senescence in NPCs and degradation of IVD.

In this study, we proposed that hypoxic umbilical cord mesenchymal stem cell-derived exosomes (hyp-UCMSC-exos) exhibit enhanced potential compared to normoxic UCMSC-exosomes (UCMSC-exos) in alleviating and reversing NPC senescence, as evidenced by *in vitro* experiments. However, the conventional method of injecting exosomes into the IVD may result in leakage, leading to reduced therapeutic efficacy. Repeated injections of exosomes are impractical in clinical practice. Therefore, alternative strategies must be explored to effectively deliver exosomes and enhance their therapeutic potential for the treatment of IVDD.

An ideal delivery platform for exosomes should preserve their bioactivity and replenish the ECM for IVD. Hydrogels composed of small bioactive molecules and/or hydrophilic polymers are ideal scaffolds for tissue engineering using exosomes. Han *et al*. suggested that a silk fibroin hydrogel laden with exosomes derived from stem cells could prevent ageing-induced vascular dysfunction [15]. Jin *et al*. found that hydrogel microspheres laden with young exosomes secreted by stem cells from human exfoliated deciduous teeth could restore the impaired function and reparative capacity of ageing tendon stem/progenitor cells [16]. Research remains limited on using exosome-loaded hydrogels to reduce IVD cell senescence in disk regeneration.

In this study, we investigated the therapeutic potential of hyp-UCMSC-exos for rejuvenating senescent NPCs. To deliver hyp-UCMSC-exos, we used an injectable biocompatible hydrogel composed of hyaluronic acid methacryloyl (HAMA). Our *in vivo* experiments demonstrated that the HAMA/hyp-UCMSC-exos system effectively reversed the senescence of NPCs, thereby alleviating IVD degradation.

## Materials and methods

### NP samples isolation and collection

NP tissues were collected from 10 patients with idiopathic scoliosis or IVDD to extract normal and degenerated NPCs. The patients’ ages were 43.7 ± 23.5 (range 17–72 years). The experiments involving human participants were approved by the Medical Ethics Committee of the First Affiliated Hospital of the Air Force Medical University in Xi’an, China (approval number: KY20243135-1). Written informed consent was obtained from all participants. NP tissues were identified using a stereotaxic microscope. A combination of 0.2% pronase and 0.025% collagenase P was used to digest the NP tissues. The NPCs were cultured in DMEM/F12-based culture medium containing 10% fetal bovine serum and 1% penicillin/streptomycin.

### Extraction of UCMSC-exos

UCMSCs were purchased from Procell (Wuhan, China) and cultured in MEM-α medium containing 10% FBS. The cells were cultured in a 37°C humidified chamber with 5% CO_2_, 21% O_2_ or 1% O_2_. Once a 95% confluency was reached, the medium was replaced with 10% exosome-free fetal bovine serum (Gibco, USA). After 48 h, the medium was collected and exosomes were extracted using gradient centrifugation. Briefly, the medium was centrifuged at 300 × *g* for 10 min, 2000 × *g* for 20 min, 10 000 × *g* for 30 min, and finally, at 100 000 × *g* for 70 min, all at 4°C. The resulting exosome pellets were, then, resuspended in phosphate-buffered saline (PBS) and stored at −80°C.

### Characterization of exosomes

Nanoparticle tracking analysis (NTA) and transmission electron microscopy (TEM) were used to assess the size and morphology of exosomes. Surface markers of exosomes (TSG101, CD9 and CD63) were detected using western blotting. Additionally, the PKH26 dye was used to label exosomes and facilitate the detection of cell phagocytosis. A confocal microscope (FV1000; Olympus) was used to capture images.

### miRNA sequencing of hyp-UCMSC-exos

The RNAiso for Small RNA kit (Takara, Japan) was used to extract RNA from hyp-UCMSC-exos samples. Subsequently, small RNAs were reverse transcribed to generate a cDNA library with initial trimming to a size of 18–30 nt. Gene Denovo Biotechnology Co. sequenced the cDNA using the Illumina HiSeq™ 2500 platform. Raw reads obtained from the sequencing were subjected to filtration, alignment and miRNA identification. Additional analyses included the evaluation of miRNA expression patterns through principal component analysis, grouping of similar miRNA expression patterns using clustering methods, identification of differentially expressed miRNAs, prediction of target genes and enrichment analysis of these genes. Differentially expressed miRNAs were confirmed based on a fold change surpassing 1.5 and a *Q*-value < 0.001. Distinct thresholds were established for upregulated and downregulated miRNAs.

### Preparation of HAMA hydrogel

HAMA hydrogel was obtained from Engineering for Life (EFL-HAMA-150K, Suzhou, China). One millilitre of HAMA solid (0.005 g) was weighed to prepare the hydrogel. Subsequently, 1 ml of the photoinitiator (0.25% w/v) was added, and the sample was stirred for 30 min. To eliminate air bubbles, the mixture was rotated at 5000 rpm. Then, a needle filter of 0.22 μm was used to filter bacteria. Finally, the HAMA hydrogel was cured by exposing it to blue light (UV405 nm, 30 mW/cm^2^) for 20 s.

### Characterization of HAMA hydrogel physicochemical structure

The chemical structure of the HAMA hydrogel was investigated using Fourier-transform infrared spectroscopy with wavenumbers ranging from 4000 to 700 cm^−1^.

### Characterization of HAMA hydrogel morphology

The morphology of the HAMA hydrogel was examined using scanning electron microscopy (SEM). Freeze drying was used to dehydrate the HAMA hydrogel in a freeze-dryer. Subsequently, the sample was affixed to a conductive gel and coated with a thin layer of gold. An SEM instrument (FEI, USA) was used to capture high-resolution SEM images.

### Characterization of the self-healing ability and rheological effects of HAMA hydrogel

The self-healing ability and rheological properties of the HAMA hydrogels were evaluated using a TA rheometer (DHR-2). The measurements included the evaluation of the critical strain and determination of the storage and loss moduli of the hydrogel. The self-healing ability of the hydrogel was assessed by subjecting it to an alternating strain mode with a step sweep.

### Characterization of exosome release from HAMA-exo hydrogel

To evaluate exosome release, we used a micro bicinchoninic acid protein assay kit (Beyotime, China). Briefly, we combined 100 μl of PBS with HAMA-exo hydrogel in the chamber of an 8 μm Corning 24-well plate. Fresh PBS was used to replace the supernatant at specific time intervals, and exosome release was measured.

### Detection of the senescent phenotype

To investigate the potential anti-senescence properties of hyp-UCMSC-exos, degenerated NPCs were incubated with a medium containing hyp-UCMSC-exos for 3 and 7 days. The presence of senescent cells was evaluated using the senescence-associated β-galactosidase (SA-β-Gal) kit (Beyotime). Briefly, the cells were treated with fixation buffer (pH 6) for 20 min and then were co-cultured with SA-β-Gal for 12–16 h. A positive signal, indicated by blue staining, was observed under a microscope.

### Reverse transcription quantitative polymerase chain reaction analysis

RNA was extracted from NPCs harvested at 3 and 7 days using RNAiso (Takara, Shiga, Japan), following the manufacturer’s protocols. The senescence-related genes were determined by utilizing GAPDH as the internal reference. The primer sequences are listed in Supplementary Table S1.

### Immunofluorescence staining

The cells were treated with 4% paraformaldehyde for 20 min. The samples were sealed with Triton-X100 and bovine serum albumin. Then, the sections underwent overnight incubation with primary antibodies targeting p21 and 5-ethynyl-2'-deoxyuridine (EdU), along with a subsequent 1 h incubation with secondary antibodies. The primary antibodies used were against p53 (1:1000; Abcam) and EdU (1:1000; CST). Finally, the nuclei were stained with DAPI, and images were acquired using a confocal microscope.

### Western blotting

Briefly, protein separation was achieved by SDS-PAGE, followed by incubation with primary antibodies. The following primary antibodies were used: CD9 (1:1000; Abcam), CD63 (1:1000; Abcam), TSG101 (1:1000; Abcam), p53 (1:1000; Abcam), MMP13 (1:1000; Abcam) and anti-GAPDH (1:1000; CST). After washing, the membranes were incubated with secondary antibodies. The band density analysis used a Bio-Rad imaging system (Bio-Rad, CA).

### Immunohistochemistry analysis

The NP tissues were fixed in a solution containing paraformaldehyde and sucrose. Subsequently, the sample was sliced into 7-μm thick sections. The primary antibodies used were aggrecan (1:400; Proteintech), Col2A1 (1:400; Proteintech), P16 (1:50; Abcam) and P21 (1:200; Abcam). DAB solution (GeneTech, China) was used to visualize the staining, followed by counterstaining with haematoxylin. Microscopic imaging was employed followed by image analysis using ImageJ software.

### Proliferation of NPCs

The CCK-8 (Beyotime) was used to measure cell proliferation. Briefly, after co-culturing the reagent with cells for 1, 3, 5 and 7 days, the absorbance of the samples at 450 nm was determined using a BioTek enzyme-labelling instrument.

### Animal model of IVDD

An IVDD model was established in Sprague–Dawley rats using needle puncture as previously described [17]. The animal experiments were approved by the Animal Research Committee of The Fourth Military Medical University (approval number: IACUC-20230655) and were conducted following the guidelines of the Institutional Ethics Review Board of Xijing Hospital. The rats were anesthetized using Hypnorm (0.3 ml/kg) and Dormicum (5 mg/kg) via intraperitoneal injection. After identifying the location of the tail caudal spine and separating the muscle tissue, an 18-gauge sterile needle was inserted into the centre of the disk at a depth of 5 mm. The needle was rotated 360° and held in place for 30 s. After injecting 3 μl of the indicated substance, rats underwent surgery and were randomly divided into four groups: normal control group, degenerative control group (DC group), hydrogel injection group (HA group) and hydrogel plus hyp-UCMSC-exos injection group (HA+EXO group). Histological evaluation and imaging of the caudal spine were performed at 4 and 8 weeks postoperatively.

### Radiology evaluation

X-ray fluoroscopy (GEXR650, USA) and micro-computed tomography (micro-CT; PerkinElmer, USA) were used to assess the IVD height. Radiographs of the caudal vertebrae were taken before surgery and at 4 and 8 weeks after surgery at the Co7/8 level. The disk height index (DHI) was calculated using ImageJ software and a previously established technique [18]. Additionally, the percentage change in the DHI (%DHI) was determined by comparing the postoperative DHI with the preoperative DHI and expressing it as a percentage. Specifically, %DHI was calculated as (postoperative DHI/preoperative DHI) × 100. CTvox software was used to generate both 3D and 2D reconstructions. Subsequently, a magnetic resonance scanner was used to capture spine images. To ensure accurate results, the protocol parameters were adjusted as follows: repetition time of 3000 ms using the spin-echo technique and echo time of 90 ms.

### Histological evaluation

Disks were collected 4 and 8 weeks after needle puncture. Subsequently, H&E staining was performed. IVD degeneration was evaluated using a previously established grading scale [19]. Two unbiased observers assessed the extent of IVDD without prior knowledge of the samples. A histological score of 5 points indicated a normal IVD, whereas a score ranging from 6 to 11 points indicated moderate IVD degeneration. In contrast, a score between 12 and 14 indicated severe IVD degeneration.

### Statistical analyses

Statistical analyses were performed using GraphPad Prism 8.3.0 software. Comparisons between two groups were performed using the Student's *t*-test, while one-way or two-way ANOVA tests were used for more than two groups. The significance level was set at *P* < 0.05. All data were expressed as mean ± standard error of the mean (SE). The graphical abstract was created using FigDraw (www.figdraw.com).

## Results and discussion

### Identification and extraction of normal and senescent NPCs

**Figure 1.**
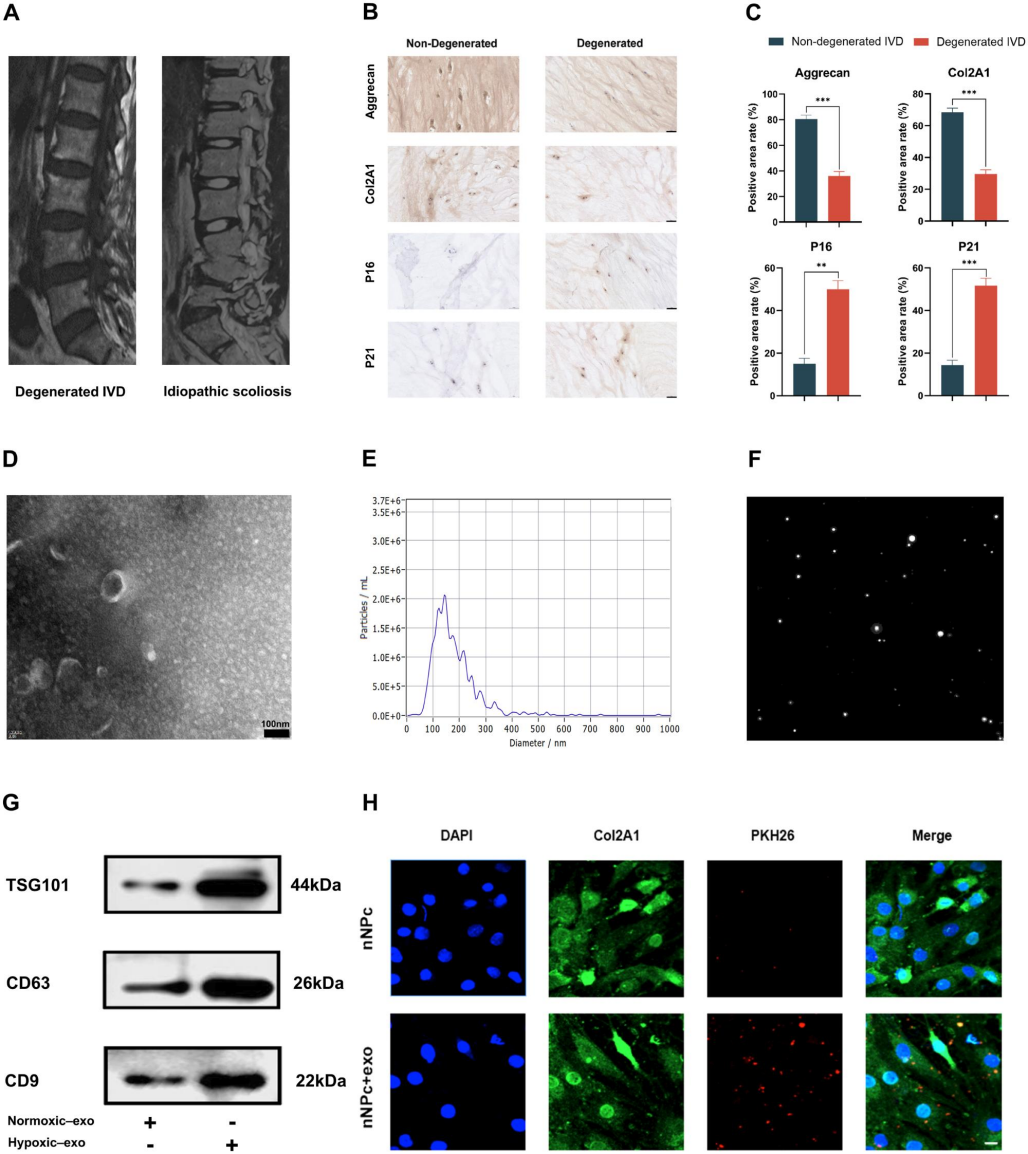
Extraction and characterization of NPCs and UCMSC-exos. (**A**) Representative images (magnetic resonance imaging) of degenerated IVD and idiopathic scoliosis in patients. (**B**) Representative images of immunohistochemistry for aggrecan, Col2A1, P16 and P21 in nondegenerated and degenerated NP tissues. (**C**) Quantitative analysis of positively stained areas for aggrecan, Col2A1, P16 and P21 from data shown in b (*n* = 3). (**D**) Representative TEM image of exosomes (scale bar, 100 nm). (**E**) Particle size distribution of exosomes assessed using NTA. (**F**) Representative image for nanoparticle tracking measurements under flow conditions. (**G**) Western blotting analysis of TSG101, CD63 and CD9 in normoxic- and hypoxic-exos. (**H**) Representative images of immunofluorescence staining of PKH26-labeled-exos incubated with NPCs (scale bar, 20 μm). Data are expressed as the mean ± SE. **P < 0.01, ***P < 0.001.

The T2-weighted magnetic resonance imaging (MRI) scans of patients with idiopathic scoliosis and IVDD are shown in Figure 1A. Immunohistochemical staining using aggrecan and Col2A1 was performed to confirm the difference in the degree of degeneration between normal and degenerated NP tissues (Figure 1B and C). The results showed a reduction in the expression of aggrecan and Col2A1 with the aggravation of IVDD, thus, confirming the successful extraction of normal and degenerated NP tissues. Additionally, P16 and P21, which are well-known markers of senescence, were used to evaluate the degree of senescence in NP (Figure 1B and C). Interestingly, these markers were scantly expressed in normal NP tissue but exhibited increased expression in degenerated NP tissues. These findings provide additional evidence for the correlation between NP cell senescence and degeneration of NP tissues and IVD. Normal and senescent NP cells were isolated from NP tissues and cultured for subsequent experiments.

### Characterization of hyp-UCMSC-exos and their internalization effect

Hypoxic preconditioning of cells enhances the secretory activity and anti-inflammatory effects of exosomes [20–22]. In this study, we collected hypoxia-induced umbilical cord mesenchymal stem cell-derived exosomes (hyp-UCMSC-exos) by ultracentrifugation. Hyp-UCMSC-exos were characterized using TEM and NTA. The TEM results demonstrated that hyp-UCMSC-exos exhibited the typical cup-shaped morphology of exosomes (Figure 1D) with a concave side [23]. NTA revealed that the diameter of hyp-UCMSC-exos ranged from 30 to 200 nm (Figure 1E and F). Additionally, western blotting experiments suggested that exosomes derived from UCMSCs under normoxic (nor-UCMSC-exos) or hypoxic conditions expressed the typical markers of exosomes: CD9, CD63 and TSG101 (Figure 1G). Interestingly, hyp-UCMSC-exos showed higher expression levels of these exosomal markers than did the nor-UCMSC-exos, suggesting an increased quantity of exosomes under hypoxic conditions. Furthermore, co-culture experiments using PKH26-labeled hyp-UCMSC-exos and NPCs demonstrated the internalization of hyp-UCMSC-exos by NPCs (Figure 1H).

### Sequencing and analysis of miRNAs in hyp-UCMSC-exos

**Figure 2.**
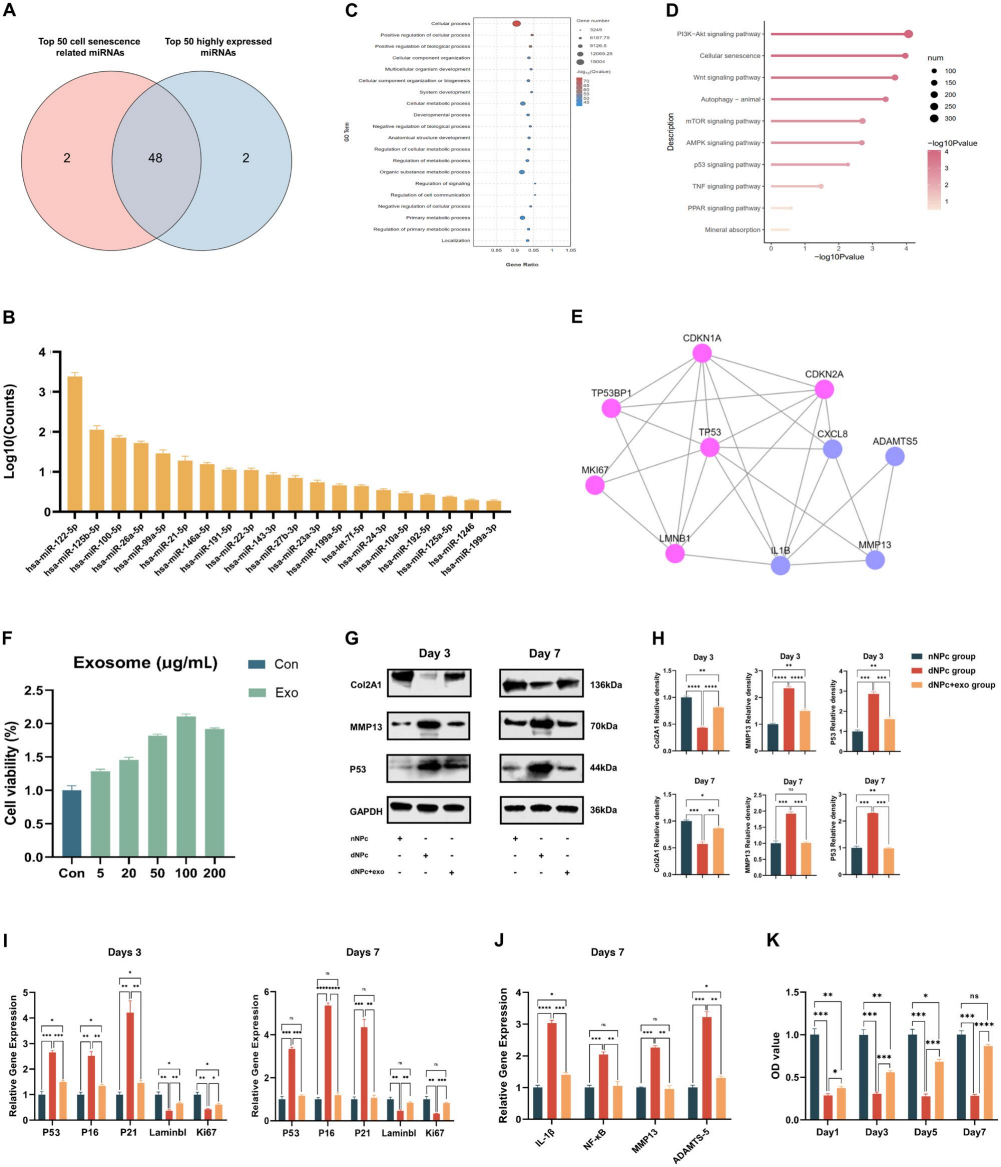
Ageing NPCs are rejuvenated by hyp-UCMSC-exos. (**A**) Venn diagram showing the screening process for identifying miRNAs associated with cell senescence and highly expressed in hyp-UCMSC-exos. (**B**) miRNAs highly expressed in hyp-UCMSC-exos and related with cell senescence (*n* = 3). (**C**) GO analysis conducted to predict the signalling pathway of hyp-UCMSC-exos. (**D**) KEGG analysis conducted to predict the signalling pathway of hyp-UCMSC-exos. (**E**) Interaction network diagram showing the p53 and SASP factor genes. (**F**) Senescent NPC viability after incubation with different concentrations of exosomes (*n* = 3). (**G**) Western blotting analysis of Col2A1, MMP13 and P16 in NPCs cultured with or without hyp-UCMSC-exos for 3 or 7 days. (**H**) Quantitative analysis of Col2A1, MMP13 and P16 expression in NPCs (*n* = 3). (**I**) RT-qPCR analysis of p53, P16, P21, lamin B1 and Ki67 expression in NPCs cultured with or without hyp-UCMSC-exos for 3 and 7 days (*n* = 3). (**J**) RT-qPCR analysis of IL-1β, NF-κB, MMP13 and ADAMTS-5 expression in NPCs cultured with or without hyp-UCMSC-exos for 7 days (*n* = 3). (**K**) NPC proliferation quantified using the CCK-8 assay (*n* = 3). Data are expressed as the mean ± SE. *P < 0.05, **P < 0.01, ***P < 0.001, ****P < 0.0001; ns, nonsignificant difference.

Sequencing of hyp-UCMSC-exos revealed that miRNAs associated with senescence were arranged in a descending manner. An intersection was performed between the top 50 miRNAs and the most highly expressed miRNAs (Figure 2A). Furthermore, 48 miRNAs that were highly expressed in hyp-UCMSC-exos and may be related to cell senescence were quantified and partly shown in Figure 2B. Extensive Gene Ontology (GO) and Kyoto Encyclopedia of Genes and Genomes (KEGG) analyses were performed to investigate the interconnected signalling pathways regulated by these miRNAs in hyp-UCMSC-exos. GO analysis demonstrated that the target genes of these miRNAs exhibit active involvement in ‘cell processes’ and ‘cellular metabolic processes’, and related processes (Figure 2C), which can contribute to the rejuvenation of senescent NPCs that have lost their proliferative ability. Additionally, KEGG pathway analysis indicated that the target genes of these miRNAs play intricate roles in pathways associated with senescence, such as the p53, Wnt and mTOR signalling pathways (Figure 2D) [24–26]. The top 10 miRNAs with the highest counts were further filtered. The top 400 target genes of these miRNAs were predicted and used to construct an interaction network (Supplementary Figure S1). Visualization of the network emphasized the central role of the TP53 (p53) gene within the entire network. The p53 pathway plays a vital role in cellular ageing. This pathway reacts to different internal and external stress signals that influence the mechanisms controlling cellular division and DNA replication [27]. Gene interaction mapping of SASP relative genes (IL1B, ADAMTS5, MMP13 and CXCL8) and p53 relative genes (TP53, TP53BP1, CDKN1A, LMNB1, CDKN2A and MKI67) further demonstrated that the p53 gene could influence the expression of cell senescence-related genes (Figure 2E). By integrating the sequencing outcomes, we can infer that hyp-UCMSC-expos may mediate the p53 signalling pathway, thus, involving the cellular senescence process.

### Rejuvenation of the ageing phenotype of NPCs by hyp-UCMSC-exos

Senescence is characterized by a decrease in anabolic metabolism, an increase in catabolic metabolism and an inflammatory response, resulting in the formation of a malignant microenvironment surrounding NPCs [28]. Previous studies have shown that UCMSC-exos can reverse cell senescence in various organs and tissues [29, 30]. However, no research has been conducted on the effect of hyp-UCMSC-exos on NPCs. Different concentrations were co-cultured with senescent NPCs to determine the appropriate concentration of hyp-UCMSC-exos required for NPC rejuvenation. As shown in Figure 2F, the viability of NPCs increased in a concentration-dependent manner when co-cultured with hyp-UCMSC-exos. However, when the exosome concentration exceeded 100 μg/ml, the increase in cell viability plateaued. Therefore, a concentration of 100 μg/ml was used for further experiments. The CCK-8 assay results indicated that co-culturing NPCs with hyp-UCMSC-exos significantly enhanced their metabolic activity (Figure 2K).

**Figure 3.**
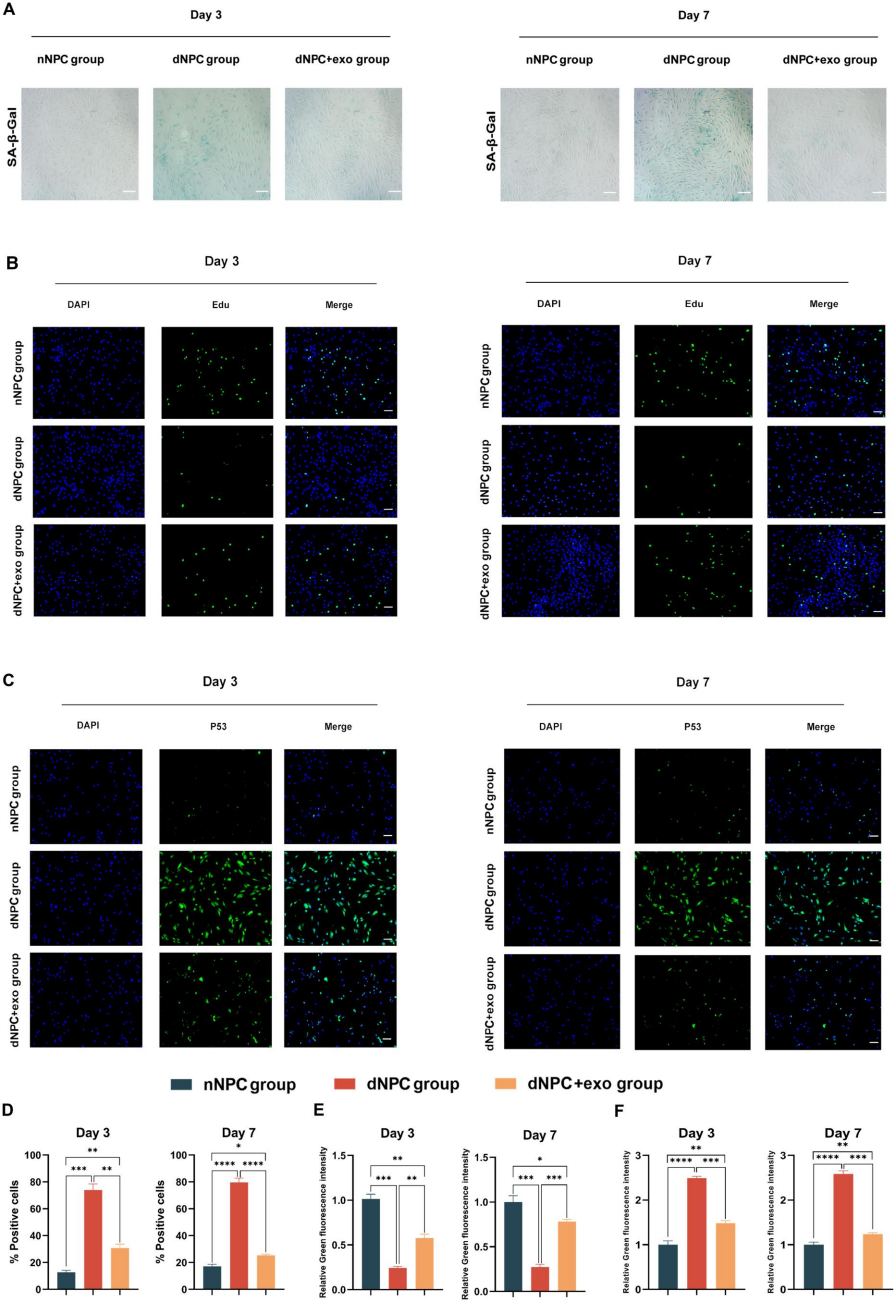
Ageing NPCs are rejuvenated by hyp-UCMSC-exos. (**A**) Representative images of SA-β-Gal staining in NPCs cultured with or without hyp-UCMSC-exos for 3 or 7 days (scale bar, 200 μm). (**B**) Representative images of immunofluorescence staining for EdU in NPCs cultured with or without hyp-UCMSC-exos for 3 or 7 days (scale bar, 100 μm). (**C**) Representative images of immunofluorescence staining for p53 in NPCs cultured with or without hyp-UCMSC-exos for 3 or 7 days (scale bar, 100 μm). (**D**) Quantitative analysis of SA-β-Gal-positive NPCs (*n* = 3). (**E**) Quantitative analysis of EdU-positive NPCs following culture with or without hyp-UCMSC-exos for 3 or 7 days (*n* = 3). (**F**) Quantitative analysis of p53-positive NPCs following culture with or without hyp-UCMSCs-exo for 3 or 7 days (*n* = 3). Data are expressed as the mean ± SE. *P < 0.05, **P < 0.01, ***P < 0.001, ****P < 0.0001. nNPC, normal NPCs; dNPC, degenerated NPCs.

Studies have shown that anabolic homeostasis of the ECM is impaired during cell senescence [31, 32]. Additionally, senescent cells can release SASP factors, including MMP13 and other inflammatory cytokines [33, 34]. Therefore, Col2A1 and MMP13 were used as markers to detect ECM and SASP expression in the degenerated NPCs after co-culture with hyp-UCMSC-exos. Western blot analysis on days 3 and 7 demonstrated a significant reversal in ECM degradation (Col2) and SASP expression (MMP13) in the group treated with hyp-UCMSC-exos compared with that in the degenerative NPC group without treatment (Figure 2G and H). Furthermore, the expression of the senescence marker P16 was significantly downregulated. p53, P16 and P21 are well-established markers that promote cell ageing, whereas Ki67 and lamin B1 are negative markers that indicate cell ageing and morphological changes [35]. Reverse transcription quantitative polymerase chain reaction (RT-qPCR) analysis showed that hyp-UCMSC-exos increased the expression of lamin B1 and Ki67 while decreasing the expression of p53, P16 and P21 (Figure 2I). These genetic effects became more apparent as the number of days of co-culture increased. Additionally, the inflammatory cytokines IL-1β, NF-κB, MMP13 and ADAMTS-5 were downregulated after co-culturing with hyp-UCMSC-exos (Figure 2J). Furthermore, on days 3 and 7, the ageing NPCs showed high enzymatic activity of SA-β-Gal. However, adding hyp-UCMSC-exos to the NPC culture significantly decreased SA-β-Gal secretion (Figure 3A). Immunofluorescence staining also demonstrated a decrease in the expression of the important senescent gene p53 and an increase in EdU staining following hyp-UCMSC-exo treatment (Figure 3B–F). Overall, the analysis of senescence markers showed that hyp-UCMSC-exos reduced senescent and inflammatory gene expression, thereby enhancing the proliferation ability, cell activity and ECM secretion of NPCs.

### Synthesis and characterization of exo-loaded hydrogel

**Figure 4.**
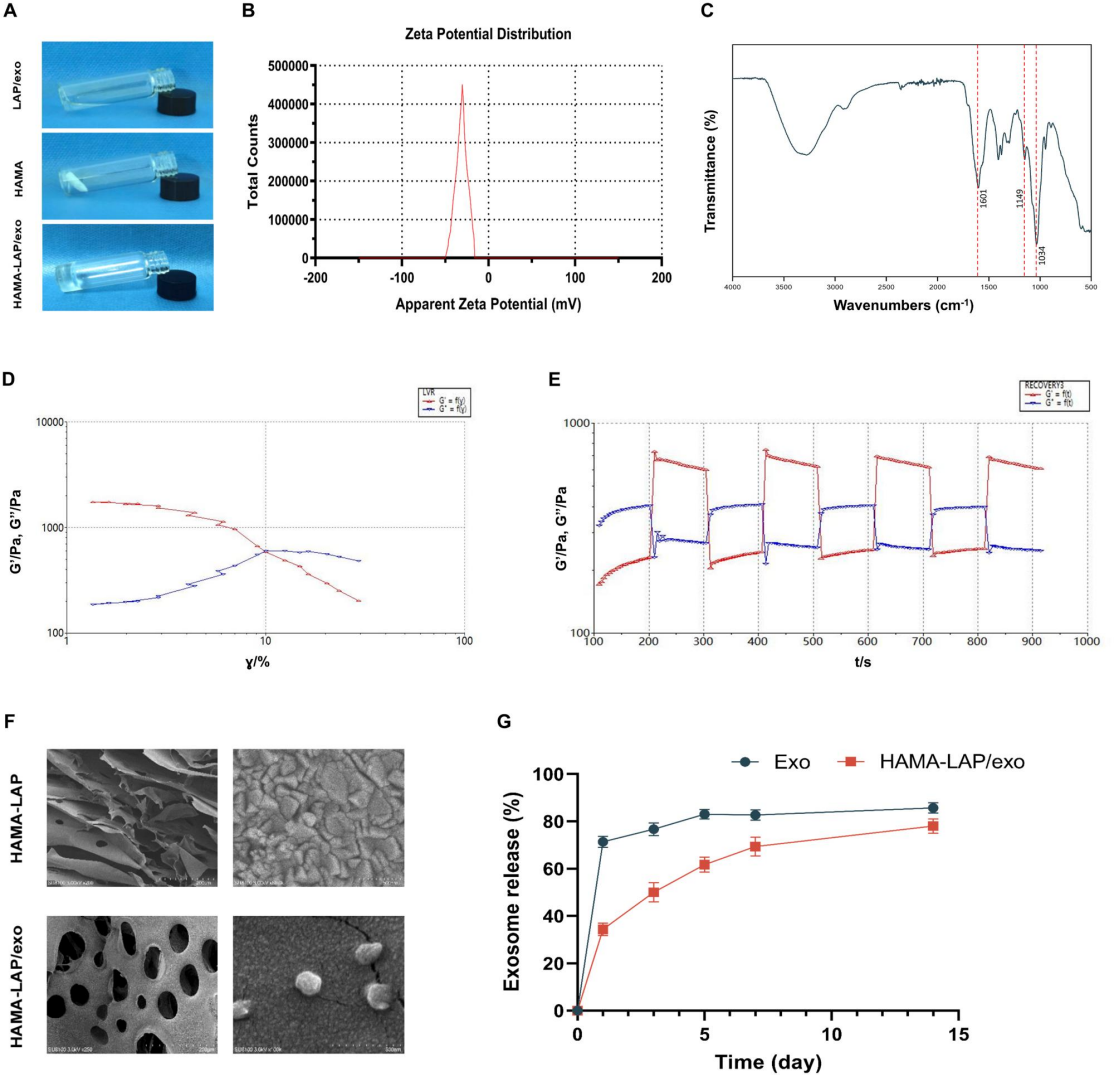
Synthesis and characterization of exo-loaded hydrogel. **(A**) Photographs showing the formation of HAMA-LAP/exos after mixing HAMA with LAP/exos. (**B**) Zeta potential of exosomes. (**C**) FT-IR spectra of the HAMA hydrogel. (**D**) Critical strain and rheology of the HAMA hydrogel. (**E**) Macroscopic self-healing behavior of the HAMA hydrogel. (**F**) Representative SEM images of the HAMA hydrogel and HAMA-LAP/exos. (**G**) Quantitative analysis of the cumulative exosome release from the HAMA hydrogel (*n* = 3). Data are expressed as the mean ± SE.

In addition to the nonspecific targeting of exosomes *in vivo*, another limitation of their clinical application is the relatively short duration of exosomes in the body. Although localized intra-IVD injection can improve the retention of exosomes, this alone is insufficient to effectively treat IVDD, as it requires long-lasting therapeutic effects. In addition, previous studies have revealed that direct injection of MSCs into the IVD might cause the formation of osteophytes [36, 37]. To solve this problem, we combined a hydrogel with exosomes to create a continuous release system. HAMA-lithium phenyl-2,4,6-trimethylbenzoylphosphinate (LAP) was formed by the homogeneous mixing of LAP/exos and HAMA, as shown in Figure 4A. The zeta potential of hyp-UCMSC-exos was -30.4 mV, which suggested that the exosome was strongly combined with HAMA (Figure 4B). Fourier-transform infrared spectroscopy was used to confirm the chemical structure of the HAMA hydrogel (Figure 4C). Subsequently, the rheological behaviour and self-healing capacity of the hydrogels were investigated. Figure 4D and E demonstrate that the HAMA hydrogel exhibited suitable mechanical properties for IVD and a desirable capacity for self-healing. SEM characterization of the hydrogel and the hydrogel loaded with exosomes is shown in Figure 4F. Both samples displayed a porous structure, whereas the exo-loaded sample exhibited a round nanostructured morphology, indicating the successful loading of exosomes onto the hydrogel matrix. Furthermore, when the exosomes were not encapsulated in the hydrogel, the release profile indicated that more than 70% of the exosomes could be detected within 24 h. In contrast, the hydrogel matrix containing the loaded exosomes showed a gradual and consistent release of exosomes over a 14 days period. After 14 days, the HAMA hydrogel released approximately 70% of the exosomes, thus, ensuring the sustained efficacy of exosomes in IVDD treatment (Figure 4G). The results of cytotoxicity tests also demonstrated that the HAMA hydrogel group exhibited cell viability values exceeding 90% with no toxic side effects, indicating that HAMA hydrogel possesses excellent biocompatibility and is suitable for biomedical material applications (Supplementary Figure S2).

### Exo-loaded hydrogel reduced NPC senescence and disc degeneration *in vivo*

**Figure 5.**
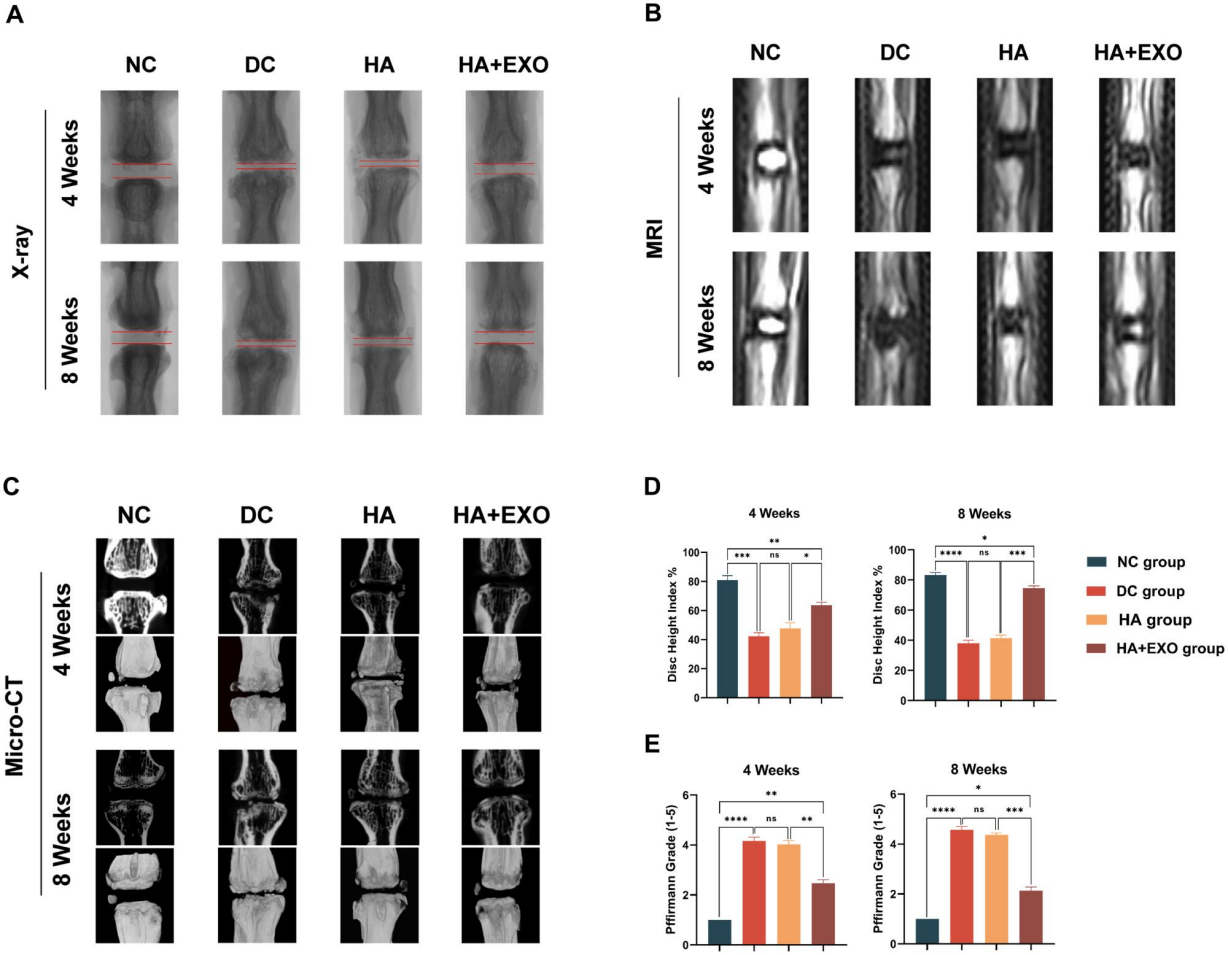
Exo-loaded hydrogel reduces NPC senescence and disc degeneration *in vivo*. (**A**) Representative X-ray images of rat spines at 4 and 8 weeks. (**B**) Representative MRI images of rat spines at 4 and 8 weeks. (**C**) Representative Micro-CT images of rat spines at 4 and 8 weeks. (**D**) Quantitative analysis of the DHI (*n* = 3). (**E**) quantitative analysis of the Pfirrmann grade (*n* = 3). Data are expressed as the mean ± SE. *P < 0.05, **P < 0.01, ***P < 0.001, ****P < 0.0001; ns, nonsignificant difference.

To evaluate the therapeutic effects of the exo-loaded hydrogel *in vivo*, PBS, hydrogel and exo-loaded hydrogel were administered to rats with IVDD. After four weeks, the disks treated with the exo-loaded hydrogel (HA + EXO group) exhibited a higher DHI than the disks treated with PBS (DC group) or hydrogel alone (HA group) (Figure 5A and D). Furthermore, at week 8, the disks in the HA+EXO group demonstrated significantly greater DHI values than those in both the DC and HA groups (Figure 5A and D). The DC group showed a substantial decrease in signal intensity and an increased Pfirrmann score, as demonstrated by T2-weighted and micro-CT MRI at weeks 4 and 8 (Figure 5B, C and E). At week 8, the disks treated with the exo-loaded hydrogel showed significantly higher signal intensity than those treated with PBS or HA. Moreover, the Pfirrmann score was significantly lower in the HA + EXO group than in the DC and HA groups. These results suggested that the exo-loaded hydrogel can alleviate disc degeneration *in vivo*.

**Figure 6.**
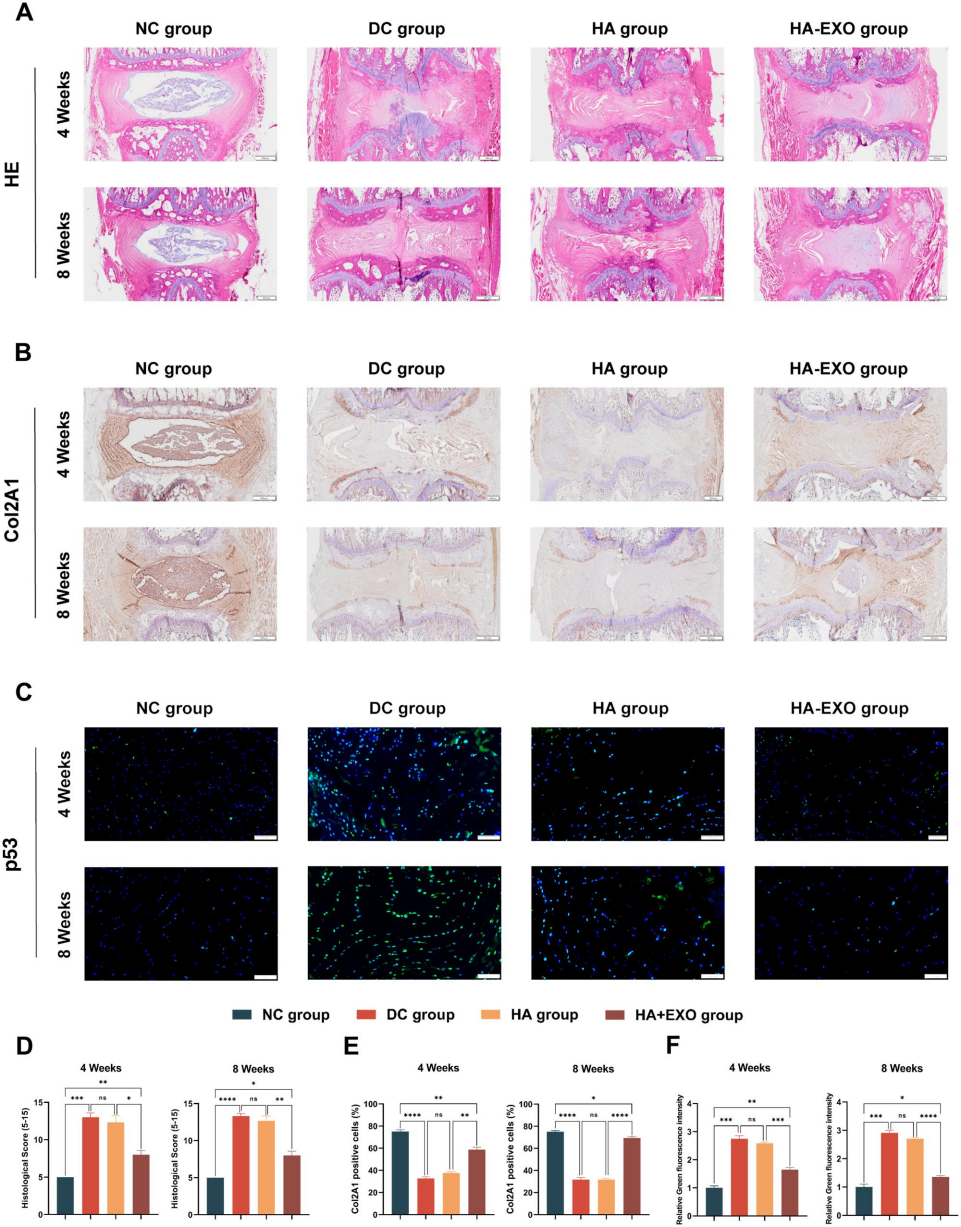
Exo-loaded hydrogel reduces NPC senescence and disc degeneration *in vivo*. (**A**) Representative images of haematoxylin–eosin staining of rat spines at 4 and 8 weeks (scale bar, 500 μm). (**B**) Representative images of immunohistochemistry on rat spines at 4 and 8 weeks (scale bar, 500 μm). (**C**) Representative images of immunofluorescence staining for p53 in rat NPCs (scale bar, 100 μm). (**D**) Quantitative analysis of histological scores of rat spines at 4 and 8 weeks (*n* = 3). (**E**) Quantitative analysis of Col2A1-positive NPCs in rats at 4 and 8 weeks (*n* = 3). (**F**) Quantitative analysis of p53-positive NPCs in rats at 4 and 8 weeks (*n* = 3). data are expressed as the mean ± SE. *P < 0.05, **P < 0.01, ***P < 0.001, ****P < 0.0001; ns, nonsignificant difference.

The results of the haematoxylin–eosin staining analysis showed that the disks treated with PBS and HA had a distorted annulus fibrosus, ruptured fibres and shrank NP (Figure 6A). In contrast, the disks treated with exo-loaded hydrogel showed a clear distinction between the annulus fibrosus and the NP. This was supported by histological scoring, which indicated that the HA + EXO group had significantly lower scores than the other groups at both the 4 and 8 week time points (Figure 6D). Additionally, immunohistochemical analysis revealed a notable upregulation of Col2A1 expression in the HA + EXO group compared with that in the DC and HA groups (Figure 6B and E). Furthermore, immunofluorescence staining demonstrated that compared with the DC and HA groups, the expression of the senescence marker p53 was significantly downregulated, which showed that HA + EXOs could reduce the senescence of NPCs (Figure 6C and F).

In summary, animal model experiments demonstrated that exo-loaded hydrogel, a sustained-release system, could potentially enhance the therapeutic efficiency of exosomes *in vivo*, suggesting that hyp-UCMSC-exos have the potential to reverse the senescence of NPCs and alleviate the degradation of IVD. Emerging evidence suggests that UCMSC-exo may represent a novel therapeutic strategy for IVDD [38, 39]. Our findings provide further evidence supporting the significant therapeutic potential of UCMSC-exo in IVDD management. However, this study has some limitations. First, the composition of UCMSC-exos is complex, including amino acids, proteins and other cargo, which may also play significant roles in the treatment of NP and require further exploration. Second, IVDD was categorized into different grades, indicating that the appropriate dose of exosomes for each grade of IVDD requires further investigation.

## Conclusions

Research has confirmed that NPC senescence plays an important role in the IVDD process. Our study suggests that hyp-UCMSC-exo can enhance the viability and proliferation of NPCs, while reducing their senescence. Additionally, a mixture of HAMA hydrogels loaded with exosomes was developed, allowing for the continuous release of exosomes over a period of time *in vivo*. The HAMA plus exosome system showed a potential to rejuvenate the abnormal metabolism of the ECM and reverse the senescence of NPCs, thereby preventing the progression of IVDD in rats. This study successfully demonstrates that the hyp-UCMSC-exo and HAMA + exosome systems could serve as promising therapeutic strategies for IVDD by reversing NPCs senescence.

## Supplementary data

Supplementary data are available at *Regenerative Biomaterials* online.

## Supplementary Material

rbaf039_Supplementary_Data

## References

[1] Sakai D , Andersson GB. Stem cell therapy for intervertebral disc regeneration: obstacles and solutions. Nat Rev Rheumatol 2015;11:243–56. 25708497 10.1038/nrrheum.2015.13

[2] Wang Y , Che M, Xin J, Zheng Z, Li J, Zhang S. The role of IL-1β and TNF-α in intervertebral disc degeneration. Biomed Pharmacother 2020;131:110660. 32853910 10.1016/j.biopha.2020.110660

[3] Huang YC , Urban JP, Luk KD. Intervertebral disc regeneration: do nutrients lead the way? Nat Rev Rheumatol 2014;10:561–6. 24914695 10.1038/nrrheum.2014.91

[4] Zhao CQ , Wang LM, Jiang LS, Dai LY. The cell biology of intervertebral disc aging and degeneration. Ageing Res Rev 2007;6:247–61. 17870673 10.1016/j.arr.2007.08.001

[5] Xu J , Shao T, Lou J, Zhang J, Xia C. Aging, cell senescence, the pathogenesis and targeted therapies of intervertebral disc degeneration. Front Pharmacol 2023;14:1172920. 37214476 10.3389/fphar.2023.1172920 PMC10196014

[6] Wiley CD , Campisi J. The metabolic roots of senescence: mechanisms and opportunities for intervention. Nat Metab 2021;3:1290–301. 34663974 10.1038/s42255-021-00483-8 PMC8889622

[7] Xu Q , Fu Q, Li Z, Liu H, Wang Y, Lin X, He R, Zhang X, Ju Z, Campisi J, Kirkland JL, Sun Y. The flavonoid procyanidin C1 has senotherapeutic activity and increases lifespan in mice. Nat Metab 2021;3:1706–26. 34873338 10.1038/s42255-021-00491-8 PMC8688144

[8] Ying L , Liang C, Zhang Y, Wang J, Wang C, Xia K, Shi K, Yu C, Yang B, Xu H, Zhang Y, Shu J, Huang X, Xing H, Li F, Zhou X, Chen Q. Enhancement of nucleus pulposus repair by glycoengineered adipose-derived mesenchymal cells. Biomaterials 2022;283:121463. 35305464 10.1016/j.biomaterials.2022.121463

[9] Ekram S , Khalid S, Bashir I, Salim A, Khan I. Human umbilical cord-derived mesenchymal stem cells and their chondroprogenitor derivatives reduced pain and inflammation signaling and promote regeneration in a rat intervertebral disc degeneration model. Mol Cell Biochem 2021;476:3191–205. 33864569 10.1007/s11010-021-04155-9

[10] Phinney DG , Pittenger MF. Concise review: MSC-derived exosomes for cell-free therapy. Stem Cells 2017;35:851–8. 28294454 10.1002/stem.2575

[11] Weng Z , Zhang B, Wu C, Yu F, Han B, Li B, Li L. Therapeutic roles of mesenchymal stem cell-derived extracellular vesicles in cancer. J Hematol Oncol 2021;14:136. 34479611 10.1186/s13045-021-01141-y PMC8414028

[12] Chew JRJ , Chuah SJ, Teo KYW, Zhang S, Lai RC, Fu JH, Lim LP, Lim SK, Toh WS. Mesenchymal stem cell exosomes enhance periodontal ligament cell functions and promote periodontal regeneration. Acta Biomater 2019;89:252–64. 30878447 10.1016/j.actbio.2019.03.021

[13] Liao CM , Luo T, von der Ohe J, de Juan Mora B, Schmitt R, Hass R. Human MSC-Derived exosomes reduce cellular senescence in renal epithelial cells. Int J Mol Sci 2021;22:13562. 34948355 10.3390/ijms222413562 PMC8709122

[14] Oh M , Lee J, Kim YJ, Rhee WJ, Park JH. Exosomes derived from human induced pluripotent stem cells ameliorate the aging of skin fibroblasts. Int J Mol Sci 2018;19:1715. 29890746 10.3390/ijms19061715 PMC6032439

[15] Han C , Zhou J, Liu B, Liang C, Pan X, Zhang Y, Zhang Y, Wang Y, Shao L, Zhu B, Wang J, Yin Q, Yu X-Y, Li Y. Delivery of miR-675 by stem cell-derived exosomes encapsulated in silk fibroin hydrogel prevents aging-induced vascular dysfunction in mouse hindlimb. Mater Sci Eng C Mater Biol Appl 2019;99:322–32. 30889706 10.1016/j.msec.2019.01.122

[16] Jin S , Wang Y, Wu X, Li Z, Zhu L, Niu Y, Zhou Y, Liu Y. Young exosome Bio-Nanoparticles restore Aging-Impaired tendon stem/progenitor cell function and reparative capacity. Adv Mater 2023;35:e2211602. 36779444 10.1002/adma.202211602

[17] Zhao X , Sun Z, Xu B, Duan W, Chang L, Lai K, Ye Z. Degenerated nucleus pulposus cells derived exosome carrying miR-27a-3p aggravates intervertebral disc degeneration by inducing M1 polarization of macrophages. J Nanobiotechnology 2023;21:317. 37667246 10.1186/s12951-023-02075-y PMC10478255

[18] Sun Z , Luo B, Liu Z, Huang L, Liu B, Ma T, Gao B, Liu ZH, Chen YF, Huang JH, Luo Z. Effect of perfluorotributylamine-enriched alginate on nucleus pulposus cell: implications for intervertebral disc regeneration. Biomaterials 2016;82:34–47. 26741882 10.1016/j.biomaterials.2015.12.013

[19] Pan Z , Sun H, Xie B, Xia D, Zhang X, Yu D, Li J, Xu Y, Wang Z, Wu Y, Zhang X, Wang Y, Fu Q, Hu W, Yang Y, Bunpetch V, Shen W, Heng BC, Zhang S, Ouyang H. Therapeutic effects of gefitinib-encapsulated thermosensitive injectable hydrogel in intervertebral disc degeneration. Biomaterials 2018;160:56–68. 29396379 10.1016/j.biomaterials.2018.01.016

[20] Jiang H , Zhao H, Zhang M, He Y, Li X, Xu Y, Liu X. Hypoxia induced changes of exosome cargo and subsequent biological effects. Front Immunol 2022;13:824188. 35444652 10.3389/fimmu.2022.824188 PMC9013908

[21] Liu W , Rong Y, Wang J, Zhou Z, Ge X, Ji C, Jiang D, Gong F, Li L, Chen J, Zhao S, Kong F, Gu C, Fan J, Cai W. Exosome-shuttled miR-216a-5p from hypoxic preconditioned mesenchymal stem cells repair traumatic spinal cord injury by shifting microglial M1/M2 polarization. J Neuroinflammation 2020;17:47. 32019561 10.1186/s12974-020-1726-7 PMC7001326

[22] Shao C , Yang F, Miao S, Liu W, Wang C, Shu Y, Shen H. Role of hypoxia-induced exosomes in tumor biology. Mol Cancer 2018;17:120. 30098600 10.1186/s12943-018-0869-y PMC6087002

[23] Jeppesen DK , Fenix AM, Franklin JL, Higginbotham JN, Zhang Q, Zimmerman LJ, Liebler DC, Ping J, Liu Q, Evans R, Fissell WH, Patton JG, Rome LH, Burnette DT, Coffey RJ. Reassessment of exosome composition. Cell 2019;177:428–45.e418. 30951670 10.1016/j.cell.2019.02.029 PMC6664447

[24] Johmura Y , Nakanishi M. Multiple facets of p53 in senescence induction and maintenance. Cancer Sci 2016;107:1550–5. 27560979 10.1111/cas.13060 PMC5132285

[25] Wu ZL , Xie QQ, Liu TC, Yang X, Zhang GZ, Zhang HH. Role of the Wnt pathway in the formation, development, and degeneration of intervertebral discs. Pathol Res Pract 2021;220:153366. 33647863 10.1016/j.prp.2021.153366

[26] Liu GY , Sabatini DM. mTOR at the nexus of nutrition, growth, ageing and disease. Nat Rev Mol Cell Biol 2020;21:183–203. 31937935 10.1038/s41580-019-0199-y PMC7102936

[27] Vogelstein B , Lane D, Levine AJ. Surfing the p53 network. Nature 2000;408:307–10. 11099028 10.1038/35042675

[28] Shi Y , Li H, Chu D, Lin W, Wang X, Wu Y, Li K, Wang H, Li D, Xu Z, Gao L, Li B, Chen H. Rescuing nucleus pulposus cells from senescence via dual-functional greigite nanozyme to alleviate intervertebral disc degeneration. Adv Sci (Weinh) 2023;10:e2300988. 37400370 10.1002/advs.202300988 PMC10477883

[29] Chang LH , Wu SC, Chen CH, Chen JW, Huang WC, Wu CW, Lin YS, Chen YJ, Chang JK, Ho ML. Exosomes derived from hypoxia-cultured human adipose stem cells alleviate articular chondrocyte inflammaging and post-traumatic osteoarthritis progression. Int J Mol Sci 2023;24:13414. 37686220 10.3390/ijms241713414 PMC10487932

[30] Yan L , Liu G, Wu X. The umbilical cord mesenchymal stem cell-derived exosomal lncRNA H19 improves osteochondral activity through miR-29b-3p/FoxO3 axis. Clin Transl Med 2021;11:e255. 33463060 10.1002/ctm2.255 PMC7805401

[31] Zhang GZ , Liu MQ, Chen HW, Wu ZL, Gao YC, Ma ZJ, He XG, Kang XW. NF-κB signalling pathways in nucleus pulposus cell function and intervertebral disc degeneration. Cell Prolif 2021;54:e13057. 34028920 10.1111/cpr.13057 PMC8249791

[32] Huang Z , Zhang N, Ma W, Dai X, Liu J. MiR-337-3p promotes chondrocytes proliferation and inhibits apoptosis by regulating PTEN/AKT axis in osteoarthritis. Biomed Pharmacother 2017;95:1194–200. 28931211 10.1016/j.biopha.2017.09.016

[33] Xu Y , Hu X, Cai J, Li Y, Zou Y, Wang Y, Xie C, Xu S, Wang Y, Zheng Y, Mahamat DA, Xu Y, Wang X, Li X, Liu A, Chen D, Zhu L, Guo J. Atractylenolide-III alleviates osteoarthritis and chondrocyte senescence by targeting NF-κB signaling. Phytother Res 2023;37:4607–20. 37380363 10.1002/ptr.7929

[34] Horváth E , Sólyom Á, Székely J, Nagy EE, Popoviciu H. Inflammatory and metabolic signaling interfaces of the hypertrophic and senescent chondrocyte phenotypes associated with osteoarthritis. Int J Mol Sci 2023;24:16468. 38003658 10.3390/ijms242216468 PMC10671750

[35] Barnes PJ , Baker J, Donnelly LE. Cellular senescence as a mechanism and target in chronic lung diseases. Am J Respir Crit Care Med 2019;200:556–64. 30860857 10.1164/rccm.201810-1975TR

[36] Zhang A , Cheng Z, Chen Y, Shi P, Gan W, Zhang Y. Emerging tissue engineering strategies for annulus fibrosus therapy. Acta Biomater 2023;167:1–15. 37330029 10.1016/j.actbio.2023.06.012

[37] Vadalà G , Sowa G, Hubert M, Gilbertson LG, Denaro V, Kang JD. Mesenchymal stem cells injection in degenerated intervertebral disc: cell leakage may induce osteophyte formation. J Tissue Eng Regen Med 2012;6:348–55. 21671407 10.1002/term.433

[38] Jia S , Yang T, Gao S, Bai L, Zhu Z, Zhao S, Wang Y, Liang X, Li Y, Gao L, Zhang Z, Gao X, Li D, Chen S, Zhang B, Meng C. Exosomes from umbilical cord mesenchymal stem cells ameliorate intervertebral disc degeneration via repairing mitochondrial dysfunction. J Orthop Translat 2024;46:103–15. 38841339 10.1016/j.jot.2023.10.004 PMC11150913

[39] Yuan X , Li T, Shi L, Miao J, Guo Y, Chen Y. Human umbilical cord mesenchymal stem cells deliver exogenous miR-26a-5p via exosomes to inhibit nucleus pulposus cell pyroptosis through METTL14/NLRP3. Mol Med 2021;27:91. 34412584 10.1186/s10020-021-00355-7 PMC8375162

