# Supplementary material for: Injectable hydrogel loaded with exosomes from hypoxic umbilical cord-derived mesenchymal stem cells alleviates intervertebral disc degeneration by reversing nucleus pulposus cell senescence
Source: Regen Biomater. 2025 May 12;12:rbaf039. doi: 10.1093/rb/rbaf039 (PMC12226454; doi:10.1093/rb/rbaf039)
Supplement: rbaf039_Supplementary_Data [file rbaf039_supplementary_data.docx]

**SUPPLEMENTARY FIGURES**


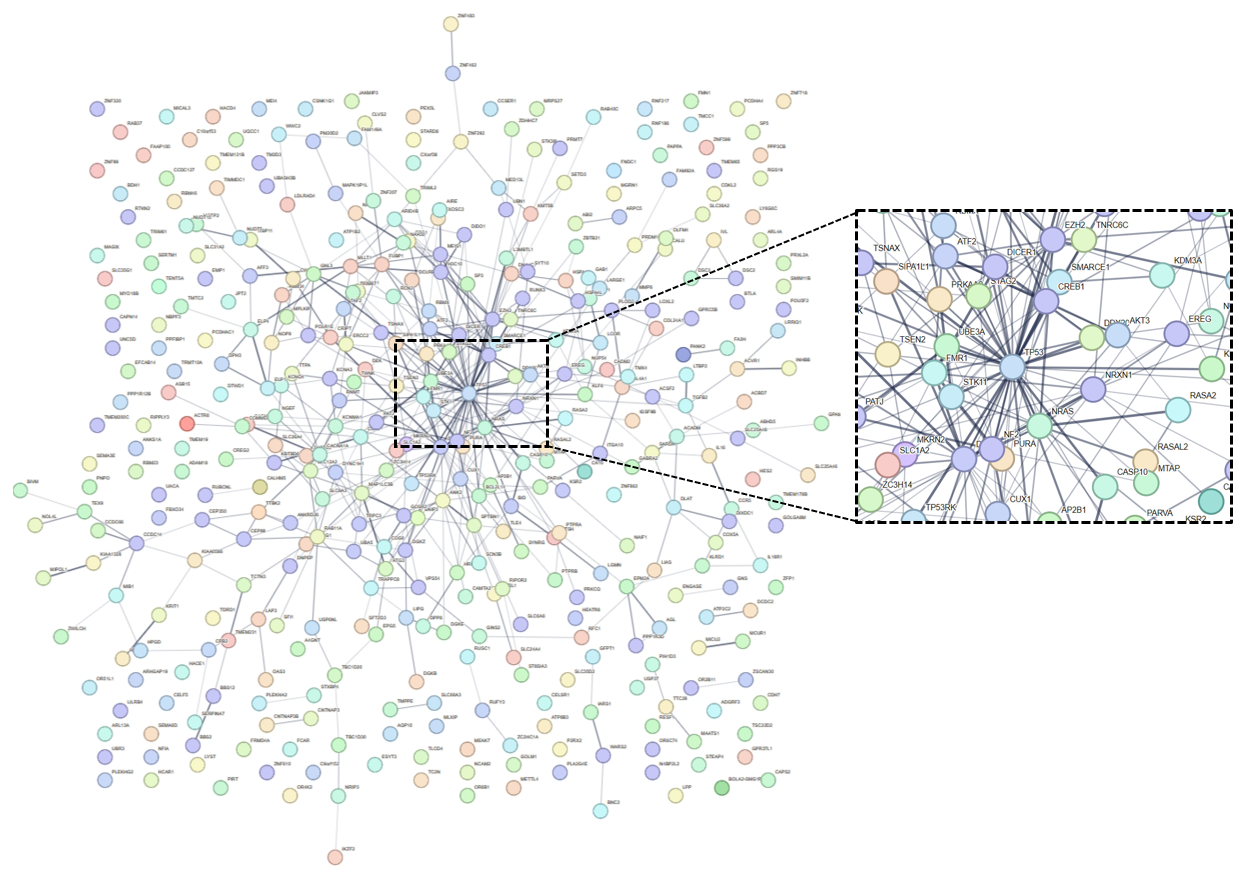


**Figure S1.** Mapping of gene interactions for the top 400 target genes based on the prediction of highly expressed miRNAs in hyp-UCMSCs-exo. The TP53 gene serves as the primary hub facilitating the connectivity of the entire network.


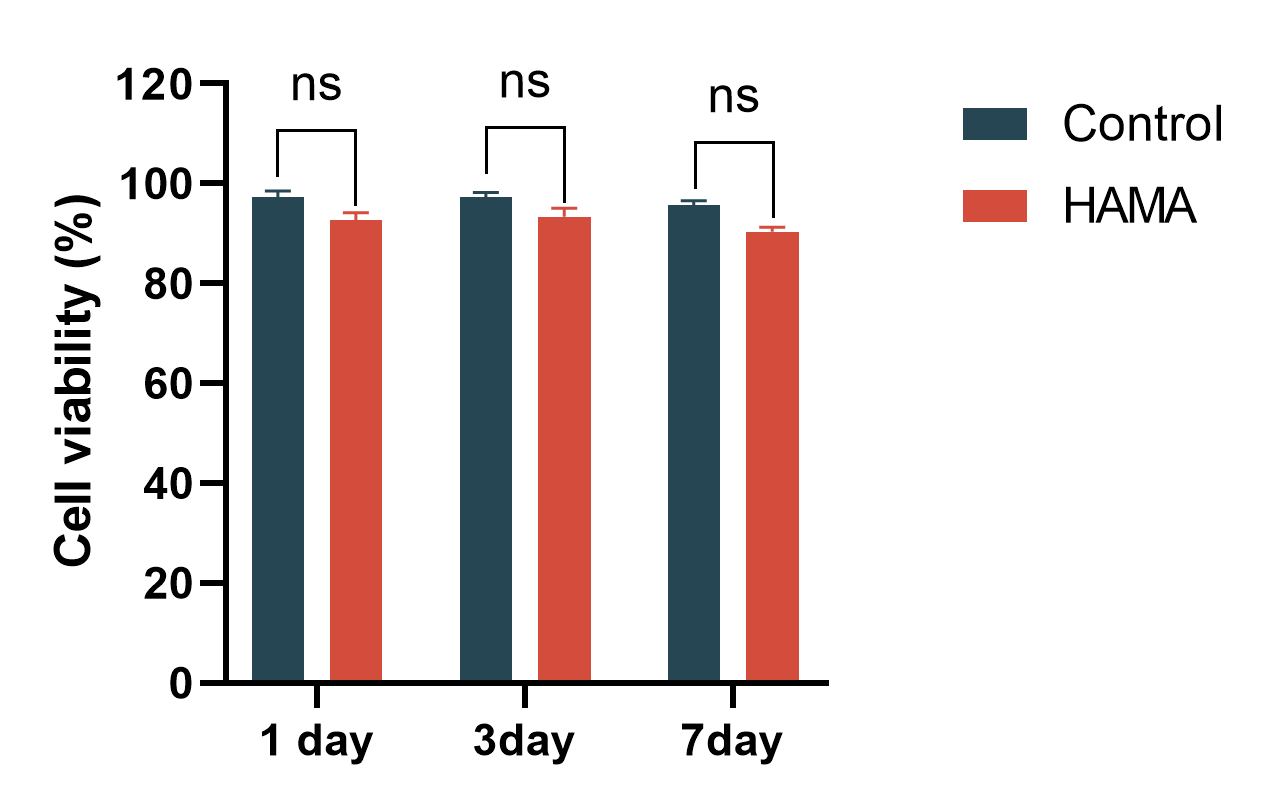


**Figure S2.** Cytotoxicity of HAMA composite hydrogel.

**SUPPLEMENTARY TABLES**

**Table S1.** The list of primers

| Gene | Forward primer | Reverse primer |
| --- | --- | --- |
| hsa P53 | CCTCAGCATCTTATCCGAGTGG | TGGATGGTGGTACAGTCAGAGC |
| hsa P16 | CTCGTGCTGATGCTACTGAGGA | GGTCGGCGCAGTTGGGCTCC |
| hsa P21 | AGGTGGACCTGGAGACTCTCAG | TCCTCTTGGAGAAGATCAGCCG |
| hsa Lamin B1 | GAGAGCAACATGATGCCCAAGTG | GTTCTTCCCTGGCACTGTTGAC |
| hsa Ki67 | GAAAGAGTGGCAACCTGCCTTC | GCACCAAGTTTTACTACATCTGCC |
| hsa IL-1β | CAGAAGTACCTGAGCTCGCC | AGATTCGTAGCTGGATGCCG |
| hsa NF-κB | TGGTAGACACGTACCGACAG | GCAGCTTGTCTCGGGTTTC |
| hsa MMP13 | CCTTGATGCCATTACCAGTCTCC | AAACAGCTCCGCATCAACCTGC |
| hsa ADAMTS-5 | TTGGCCTCTCCCATGACGAT | CGTGGTAGGTCCAGCAAACA |
